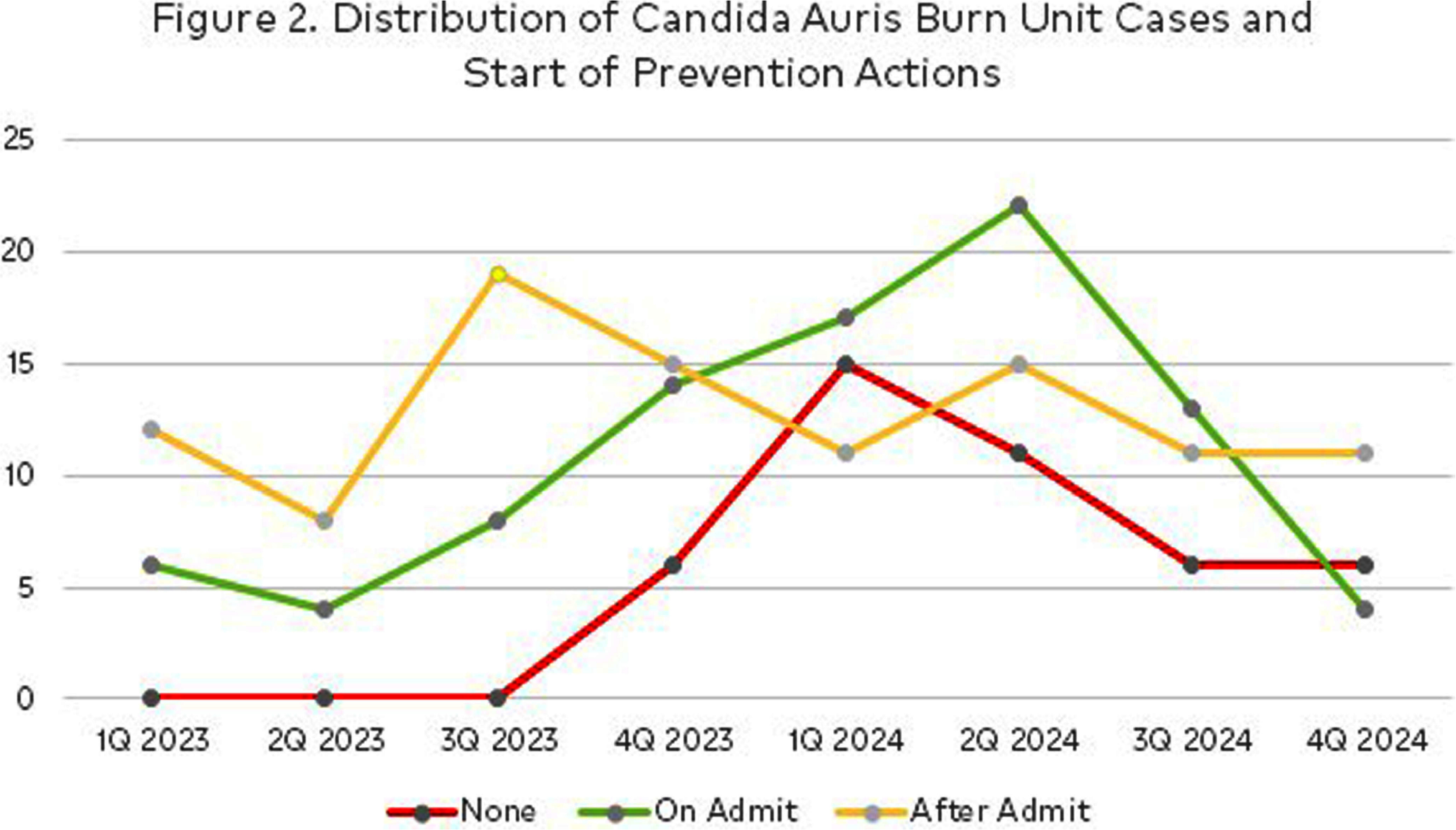# Candida auris Cluster and Mitigation in Burn Center

**DOI:** 10.1017/ash.2025.330

**Published:** 2025-09-24

**Authors:** Erik Stuckart, Casey Walker, Brooke McKie, Jane Echols, Julia Moody, Ken Sands

**Affiliations:** 1 Doctors Hospital of Augusta; 2 HCA Healthcare

## Abstract

**Background:** Candida auris (CA) is an urgent threat per CDC with rapidly increasing cases across the US. Patient rooms quickly recontaminate after daily cleaning due to skin shedding, CA persistence on environmental surfaces and resistance to surface disinfectants. Burn services experienced a sharp increase in 2023-2024 CA cases concurrently with statewide reported increases. Dry hydrogen peroxide (cDHP) is an environmental air technology augmenting daily room disinfection with activity against CA. **Investigation:** The 113 bed hospital treats complex burns and wounds as a Level 2 trauma center serving a large geographic region. Room design is single patient rooms with one semi-private ward. A total of 236 patient encounters were coded by quarter based on CA identification from clinical or surveillance cultures. cDHP was deployed in Burn ICU as of 3/2023 and nonICU as of 6/2024, with prioritization for cDHP in patients with an expected LOS of > 14 days. Surface disinfectants with CA label claim were implemented upon CA case identification. Patient skin surveillance cultures were taken upon admission burn/wound intake process and tested by the state department of health (DOH) starting in 3/2023. ATPase testing occurred as an indirect measure of cleaning and disinfection. **Findings:** Figure 1 displays the occurrence of CA burn service patients. The majority of initial positive culture sources were wound/tissue (43%) and skin surveillance (35%). Figure 2 displays when CA specific prevention actions were initiated and 63% CA cases were identified after admission. Six CA patients were in semi-private rooms. However, transmission was absent based on surveillance cultures. No statistical difference in ATPase pass/fails was found between cDHP and control room surfaces after daily cleans. In CA cases detected post discharge, all tested negative upon admission, were in single patient rooms and were more frequent within months of high CA case burdens. **Conclusions:** Surveillance testing is important for assessing burden of CA colonization, which fluctuated over this 2 year period. Despite increase in CA burden on admit, new hospital acquisition remained relatively constant Infection prevention resources shifted to observations of compliant practices. Next steps are to increase cDHP use as supplemental room disinfection, regardless of anticipated length of stay and investigate potential risks associated with silent CA acquisition identified post discharge.

**Figure:**
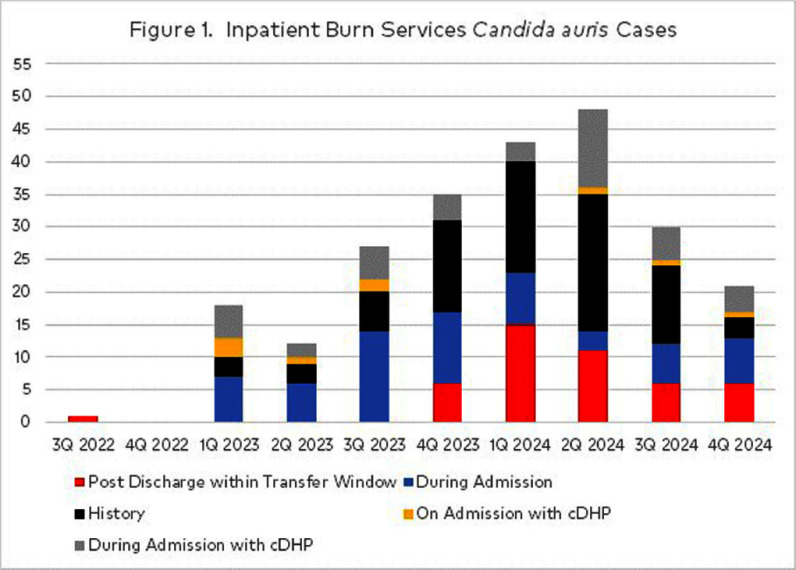

**Figure:**